# Development of Tanner Stage–Age Adjusted CDC Height Curves for Research and Clinical Applications

**DOI:** 10.1210/jendso/bvaa098

**Published:** 2020-07-17

**Authors:** Bradley S Miller, Kyriakie Sarafoglou, O Yaw Addo

**Affiliations:** 1 Pediatric Endocrinology, University of Minnesota Masonic Children’s Hospital, Minneapolis, Minnesota; 2 Department of Experimental & Clinical Pharmacology, University of Minnesota College of Pharmacy, Minneapolis, Minnesota; 3 Rollins School of Public Health, Emory University, Atlanta, Georgia

**Keywords:** Height, puberty, growth charts, ethnicity, national surveys, epidemiologic methods

## Abstract

**Background and Objective:**

Variations in normal pubertal development, pubertal disorders, and race/ethnicity can lead to differences in growth patterns and timing that are not captured by the Centers for Disease Control and Prevention (CDC) height-for-chronological age (CA_Height_) charts. Therefore, we sought to develop new Tanner stage–adjusted height-for-age (TSA_Height_) charts accounting for these differences.

**Study Design:**

Population-based Tanner staging and anthropometric data for 13 358 children age 8 to 18 years from 3 large US national surveys: National Health Examination Surveys (NHES cycle III); the Hispanic Health and Nutrition Examination Surveys (HHANES) and the third National Health and Nutrition Examination Surveys (NHANES III) were analyzed. TSA_Height_ semi-parametric models with additive age splines were used to develop smoothed TSA_Height_ curves accounting for maturation stage and calendar age.

**Results:**

As expected, the TSA_Height_ curves did not track along the respective percentile curves for the CDC 2000 CA_Height_ curves. We generated race/ethnicity–nonspecific and race/ethnicity–specific TSA_Height_ charts stratified by sex and plotted against the CDC 2000 CA_Height_ curves to account for the pubertal status differences between these models. An online calculator to adjust height for pubertal status was created.

**Conclusions:**

TSA_Height_ charts provide a much-needed tool to assess and manage linear growth for US children over the course of puberty. These tools may be useful in clinical management of children with pubertal timing variations.

The pubertal activation of the hypothalamic-pituitary-gonadal (HPG) axis is associated with several obligatory endocrine processes that synergize with other endocrine axes and impact the physical growth [1] and maturation of all body systems [2-5]. A prominent feature of adolescence is skeletal growth acceleration (“growth spurt”) and eventual deceleration of growth as adult height is reached. Up to 36 cm (or 15%) of eventual adult height can be accrued after pubertal onset [6, 7]. Although the public health interventions that improve health and nutrition of adolescents can have multigenerational benefits in terms of economic productivity, adult height and future offspring birth outcomes [8-11], most interventions focus on infants and prepubertal children. The variability in timing of pubertal maturation, rapid growth, and changes in body composition and the scarcity of tools capable of quantifying and adjusting for the effect of puberty on growth indicators may lead to less accurate and poor timing of interventions in the adolescent age group.

Lack of appropriate tools to adjust for pubertal maturation could also hinder the ability of a clinician to recognize when or whether a therapeutic intervention is needed. The major growth charts (eg, CDC 2000, WHO 2007, and UK90) used in clinical practice and auxological calculators currently available are based upon cross-sectional reference data [12-15] and only use growth parameters conditioned on chronologic age. However, when evaluating growth in clinical practice, pubertal status is heavily considered in clinical decision-making. Therefore, tools assessing “normal” or “abnormal” growth in the clinical setting that account for pubertal timing are needed. Reference tools incorporating pubertal status would be useful in order to properly categorize the growth patterns in children with normal variants of puberty (eg, early or late puberty within the normal spectrum of pubertal timing), both for clinical and research purposes. Thus, a child with late normal puberty would be less likely to be classified as short if pubertal status was not considered [16]. This is particularly important when considering how different race/ethnicity backgrounds affect the timing of puberty in children [17].

Growth curves generated from cross-sectional reference data that incorporate pubertal status would have direct clinical use in projecting adult height attainment. The ability to predict the adult height of an individual allows the clinician to assess whether a child is growing appropriately for his or her genetic potential, or whether a condition or treatment is affecting the expected height outcome. Currently, clinicians estimate the adult height for a child using the age-based height charts such as the CDC 2000 growth chronological age height (CA_Height_) charts [18], which implies that a child will stay in the same growth channel until adulthood [19]. However, this technique assumes normal growth, pubertal timing, and pubertal progression, which is not always the case. Using height charts adjusted for pubertal status, a clinician could predict a child’s adult height accurately because this method also captures inherited genetic characteristics related to timing and tempo of pubertal maturation [20]. In a previous work [16], we highlighted the inadequacy of not including pubertal timing and showed that the prevalence of shortness and tallness among Mexican American, Non-Hispanic White, and Black individuals was significantly impacted by uncaptured race/ethnicity pubertal maturation effects. We also highlighted the practical utility of Tanner stage–Height-for-Age reference charts (TSA_Height_ charts) using data from NHANES III 1988-1994, demonstrating that the use of TSA_Height_ charts could help avoid misclassification of children who have early or late puberty based upon height and age alone. The role of differences in timing of pubertal staging across the race/ethnicity groups was normalized by the TSA_Height_ adjustment method. In the current study, using a more expansive US dataset collected from 1966 to 1994 (including data from a large group of Hispanics (Hispanic HANES 1982-1984 [21], NHES cycle III 1966-1970, and NHANES III 1988-1994), we sought to develop: (a) sex and race/ethnicity–nonspecific and race/ethnicity–specific TSA_Height_ charts; (b) a TSA_Height_ Z-score calculator; and (c) TSA_Height_ reference tables for US youths ages 8 to 18 years.

## Methods

### Study population and data sources

In order to develop our TSA_Height_ charts, Z-score calculator, and references ranges, we used pooled data from 3 US cross-sectional nationally representative surveys with assessment of pubertal maturational stage from 1966 through 1994: The National Health Examination Surveys (NHES cycle III); the Hispanic Health and Nutrition Examination Surveys (HHANES 1982–1984), and the third National Health and Nutrition Examination Surveys (NHANES III). In the Hispanic HANES, Tanner stage data was available for 97.7% of the cases (2.5%, 60/2828 refusals or no Tanner stage assessment). In the NHANES 1988-1994, there were 15.0% (775/5157) refusals, or no Tanner stage assessed. Only 82.6% of children with anthropometry data had Tanner stage data.

Our cohort consisted of children aged 12 to 17 years (from NHES cycle III) or 8 to 18 years (from NHANES III) [22], the same dataset used to develop the CDC 2000 (CA_Height_) curves. Our analytic sample comprised children who had pubertal maturational status data as assessed by Tanner staging. The HHANES data were not included in the CDC 2000 (CA_Height_) curves [22]. The HHANES [21] survey comprised data from Mexican Americans (residing in 5 Southwestern US states: TX, CO, NM, AZ, and CA); Cuban Americans (in Miami-Dade County, FL) and Puerto Rican Americans (in NY, NJ, and CT) and followed the exact NHANES survey protocols. The HHANES data were also appropriately weighted to be representative of the 3 largest Hispanic American groups residing in the US at the time and assessed Tanner Staging in youth 10 to 17 years. In addition to the Mexican Americans, HHANES included data from a total of 818 participants of the HHANES cycle with nonmissing height and Tanner data—Cuban (n = 208) & Puerto Rican (n = 610) Americans—which were analyzed. Informed consent was obtained from participants (parent/guardians for minors), and all data collection was approved by the Ethics Review Board of the CDC’s National Center for Health Statistics.

### Study measures and inclusion criteria

In NHES III, HHANES, and NHANES III, standing height to the nearest 0.1 cm was measured by trained technicians using a stadiometer and following standardized protocols [23]. Pubertal status was determined following Marshall-Tanner [24, 25] criteria (Tanner Stage) by trained physicians based on secondary sexual characteristics—breast stage (girls) [20] and genital development by inspection (boys) [19] but not palpation or orchidometer assessment; the categories included Tanner Stage I (prepubertal), Tanner Stage II (early puberty), Tanner Stage III (mid-puberty), Tanner Stage IV (mid-puberty) and Tanner Stage V (late puberty/adult). We categorized participant-reported race/ethnicity into non-Hispanic White (NHW), non-Hispanic Black (NHB), Hispanic American (HIS) and “other race/ethnicities” (eg, Asian American, Native American, multiracial, mixed ancestry). We analyzed attained height and Tanner staging of US youth ages 8 to 18 years from these cohorts as a combined group and separated into ethnicity categories. We performed a test of heterogeneity in linear growth patterns among the 3 key Hispanic American ethnicities (HIS, consisting of Mexican Americans, Cuban Americans, and Puerto Rican Americans) by examining height-for-age standard deviation score (SDS) across Tanner stage and sex and found no systematic differences allowing pooled statistical analyses (results not shown). It was decided to perform a pooled analysis for all Hispanic Americans, as opposed to stratified analyses for the 3 Hispanic ethnicities. The analytic flow diagram and the data sources used in this research are displayed in the Supplementary Appendix Fig. 1 [26].

### Statistical methods

We used specialized semiparametric models to adjust attained height (somatic size) due to pubertal maturation status and chronologic age. Following this approach which we termed, *Tanner Stage Adjustment* (TSA) [16] we calculated a TSA_Height_ smoothed height percentile curves within each pubertal maturation stage and according to sex and chronologic age. The Lambda-Mu-Sigma (LMS) growth modeling technique in used in many [12-15] growth reference charts. The Box-Cox Power Exponential distribution family in GAMLSS [27, 28] with locally weighted age splines smoothing were used to generate these specialized TSA_Height_ charts. Generic race/ethnicity–nonspecific (and mixed-ethnicity), and race/ethnicity–specific (NHW, NHB, and HIS) and stratified by sex and Tanner stage TSA_Height_ charts were created. Detailed statistical and visual diagnostic tools were followed to select the best fitting model for each chart [29]. We accounted for sampling weights to generate nationally representative TSA_Height_ charts.

We conducted sensitivity analyses characterizing differences between the CDC 2000 CA_Height_ versus our TSA_Height_ subpopulations in terms of demographics (race/ethnicity and poverty based on US government-defined poverty-income ratio [PIR] [30, 31] definitions) and anthropometry. All statistical analyses were conducted in R 3.6.0 (The R Foundation for Statistical Computing and Graphics, Vienna, Austria), and data management was carried out in SAS 9.4 (SAS Institute, Cary NC, USA).

## Results

Cross-sectional data from 13 358 participants (51.1% male; 50.5% from NHES III, 15.9% from HHANES, 32.8 % from NHANES III based upon available data per Appendix Fig. 1) [26] were included in the development of the TSA_Height_ charts. The analytic sample sizes used to estimate the TSA_Height_ for the race/ethnicity–nonspecific and race/ethnicity–specific charts and by sex, were large and robust, with each unweighted count per Tanner stage ranging from 146 to 2173 observations [32] for the smoothed percentile curve estimations (Table 1) [27]. The TSA_Height_ population represents children with Tanner stage assessment and height data and includes: (a) a subset of the population used to generate the CDC 2000 Height (CA_Height_) charts (NHES III and NHANES III); and (b) the HHANES study population which was added to increase the number of children of Hispanic ethnicity. Sensitivity analyses comparing these 2 populations show that the subgroup with pubertal assessment were on average older (~1 year), taller, and had higher body mass index (BMI) than the CDC 2000 (CA_Height_) population (Table 2). The TSA_Height_ subpopulation however had higher prevalence of youths coming from poorer households (66.8% vs 47.5%).

**Table 1.** Data Sources for Tanner Stage Height-for-Age Reference Charts for US Children Ages 8 to 19 Years

Tanner Stage	Race/Ancestry Neutral Charts (Pooled^a^, N = 13 358)		Gender & Ancestry Stratified Analyses (N = 13 131)					
			Males(n = 6724)			Females (n = 6407)		
	Males (n = 6828)	Female (n = 6530)	NHW (n = 3568)	NHB (n = 1266)	HIS (n = 1890)	NHW (n = 3127)	NHB (n = 1194)	HIS (n = 1651)
I	1002	593	335	176	474	174	136	264
II^a^	1199	638	574	244	356	231	112	279
III	864	966	437	181	232	468	161	320
IV	1174	1620	685	177	290	1061	267	272
V	2589	2713	1537	488	538	1331	620	711

^a^Includes 227 “Other” race/ethnic groups (Asian, Native American, Multiracial) etc. Abbreviations: HIS, Hispanic; NHB, Non-Hispanic Black; NHW, Non-Hispanic White.

**Table 2.** Sensitivity Analyses for Characterizing Differences Between the CDC-2000 Growth vs Tanner Stage Height-for-Age Chart Populations

Characteristics	Overall			Boys			Girls		
	CDC2000	TSA-HT	*P value	CDC2000	TSA-HT	*P* value	CDC2000	TSA-HT	*P* value
	Mean/%			Mean or %			Mean or %		
Ages	11.0	11.9	<0.0001	11.05	11.93	<0.0001	11.0	11.9	<0.0001
Height	145.6	151.0	<0.0001	145.5	150.8	<0.0001	145.7	151.7	<0.0001
Weight	39.34	45.2	<0.0001^†^	38.9	44.6	<0.0001†	39.9	45.9	<0.0001^†^
BMI	18.2	19.5	<0.0001^†^	17.9	19.3	<0.0001†	18.4	19.8	<0.0001^†^
Poverty (PIR), %									
Low	47.5	66.8	<0.0001	46.3	65.4	<0.0001	48.7	68.4	<0.0001
Medium	36.4	20.7		37.2	21.4		35.7	20.0	
High	16.1	12.5		16.5	13.3		15.6	11.7	

*P values estimated from chi-square test for proportions, *t*-tests or Wilcoxon (^†^) test of medians for continuous variables. Poverty was derived from 2 variables: poverty income ratio based on US Department of Labor thresholds (PIR), when available, or reported household income.

**Figure 1.**
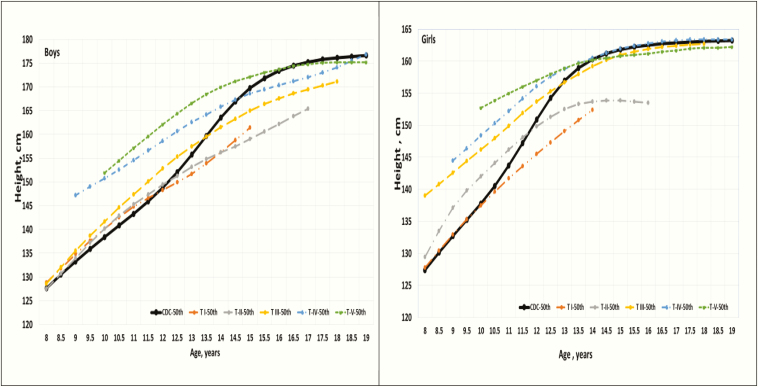
Median TSA height percentile ranges for US children in comparison to the CDC height. (race/ethnicity–nonspecific Tanner stages I-V, ages 8 to19 years, from Appendix Table 2 & 3 [26]).

Due to the cross-sectional nature of the data, it is not possible to determine age at entry into a pubertal stage, but the distribution of ages of children within each Tanner stage provides information about pubertal timing (based on *“age in-stage”*). There were race/ethnicity differences in pubertal timing across the pubertal maturation stages demonstrated by median age in each Tanner stage (Table 3). For example, median age-in-Tanner II for boys was 12.2 years for NHW, 11.5 years for NHB and 11.3 years for HIS and for girls was 11.7 years for NHW, 10.1 years for NHB and 10.9 years for HIS. In terms of population pubertal maturation tempo, only 1.7% to 4.4% of all participants were considered in “early puberty” based on their age being younger than the US published national timing estimates by Sun et al, for their sex and race/ethnicity population median age-at-entry into Tanner stage II [16, 17]. Percentages of the pubertal tempo (Appendix Fig. 2) [26] showed more “early puberty” boys in the HIS group relative to their peers (4.4% vs 3.1% NHW and 1.7% NHB; *P* ≤ 0.001). Figure 1 (from Appendix Tables 1 and 2) [26] displays race/ethnicity nonspecific normative median height percentiles according to Tanner stage and chronological ages compared with the CDC 2000 (CA_Height_) median curve. In general, linear growth trends and attained height (cm) within each Tanner stage varied markedly across age and sex relative to the CDC 2000 (CA_Height_) median trends. When accounting for pubertal status, the median linear growth curve in TSA_Height_ charts tracked closely in boys from Tanner Stage I to Tanner Stage III up to age <10.5 years. In contrast, when accounting for pubertal status in girls, their population average growth spurt occurred earlier in time as well as earlier within the pubertal maturation process. Also, the median linear growth curve in TSA_Height_ charts was highly variable across all Tanner stages until age 16 years, when the median linear growth curves seemed to converge for Tanner III to V. The median linear growth curves adjusted for Tanner stage in boys did not converge in later Tanner stages. These timing and population height variations can affect evaluation of normative growth when pubertal maturation staging is ignored. For example, the expected average height of a 10.5-year-old boy would be 142.9 cm if he is Tanner stage II; but if he is Tanner IV, his expected average height would be 152.6 cm (Appendix Table 1) [26]. Again, knowing the Tanner stage would help the clinician determine whether the child has appropriate growth for his pubertal status. Similar trends were observed in the race/ethnicity–specific TSA_Height_ tables (Appendix Tables 3-8) [26].

**Table 3.** Percentile Distribution of Ages-In-Tanner Stages Participants by Gender, Race/Ethnicity for all Participants (1966-1994, N = 13 131)

Tanner	Ages, y (Non-Hispanic White)					Ages, y (Non-Hispanic Black)					Ages, y (Hispanic Americans)				
**Males (Genitalia)**	**Min**	**25** ^**th**^	**50** ^**th**^	**75** ^**th**^	**Max**	**Min**	**25** ^**th**^	**50** ^**th**^	**75** ^**th**^	**Max**	**Min**	**25** ^**th**^	**50** ^**th**^	**75** ^**th**^	**Max**
Stage I	8.0	8.9	10.0	12.0	16.8	8.0	8.8	10.1	11.8	14.8	8.0	9.4	10.1	11.0	15.0
Stage II	8.0	10.6	12.2	13.0	16.2	8.2	10.0	11.5	12.6	15.8	8.1	10.2	11.3	12.4	16.0
Stage III	8.1	12.3	12.9	13.8	17.9	8.1	11.4	12.7	13.6	17.1	9.4	12.0	13.0	14.0	16.4
Stage IV	11.6	13.9	14.8	16.0	18.9	9.7	13.4	14.9	16.0	18.4	10.9	13.9	15.0	15.9	18.9
Stage V	12.1	15.3	16.4	17.3	19.0	11.6	15.0	16.2	17.5	19.0	12.0	15.5	16.9	17.7	19.0
**Females (Breast)**	**Min**	**25** ^**th**^	**50** ^**th**^	**75** ^**th**^	**Max**	**Min**	**25** ^**th**^	**50** ^**th**^	**75** ^**th**^	**Max**	**Min**	**25** ^**th**^	**50** ^**th**^	**75** ^**th**^	**Max**
Stage I	8.0	8.8	9.4	10.1	14.3	8.1	8.6	9.2	10.2	12.8	8.0	8.9	9.8	10.5	17.1
Stage II	8.1	10.7	11.7	12.5	15.5	8.1	9.2	10.1	11.6	14.9	8.1	10.1	10.9	11.9	16.0
Stage II	8.7	12.1	13.0	14.0	18.0	8.3	11.3	12.2	13.2	17.4	8.5	11.5	12.4	13.9	17.9
Stage IV	11.1	13.5	14.8	16.1	18.8	9.3	12.5	13.7	15.4	18.3	10.0	12.7	13.9	14.9	18.1
Stage V	11.2	14.6	16.1	17.3	19.0	10.1	14.0	15.8	17.2	19.0	11.0	14.8	16.0	17.2	19.1

**Figure 2.**
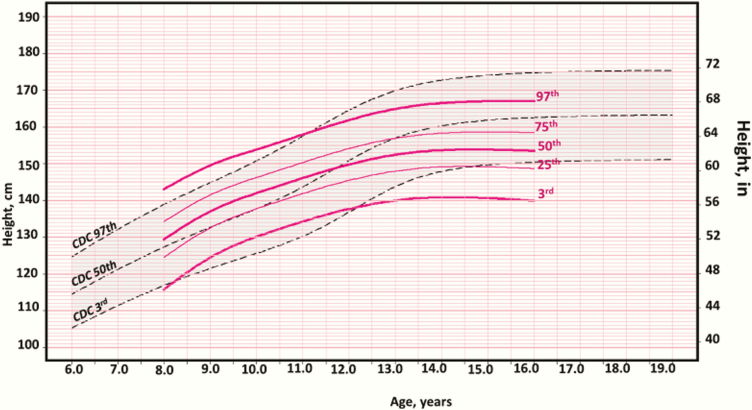
Girls race/ethnicity–nonspecific Tanner stage height-for-age percentile charts—Tanner II.

**Figure 3.**
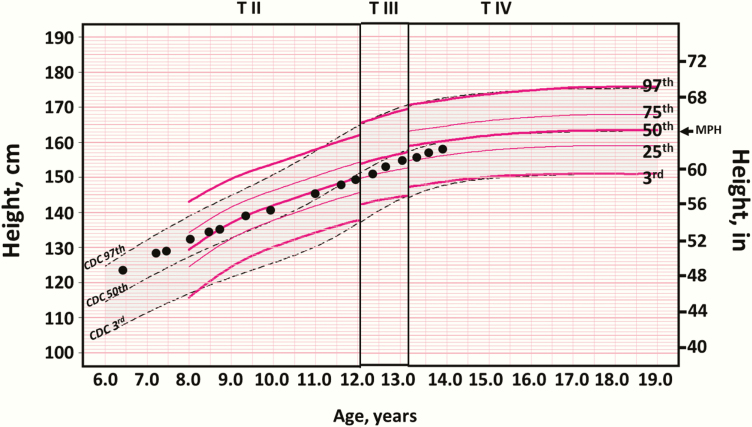
Illustration of the clinical use of the Tanner stage height-for-age chart to monitor growth during puberty suppression in a child with congenital adrenal hyperplasia and precocious puberty.

In order to develop a clinical tool for assessment of growth during the different Tanner stages we overlaid linear growth patterns as derived by TSA_Height_ method on the CDC 2000 (CA_Height_) growth charts as shown in Fig. 2 and Appendix Figs 3 to 42 (TSA_Height_ charts for Tanner I-V for both sexes) [26]. Figure 3 displays the potential clinical use of the TSA_Height_ chart for a patient with classic congenital adrenal hyperplasia with advanced bone age and growth acceleration who was treated with a gonadotropin-releasing hormone (GnRH) analogue (puberty blocker). The legend for Fig. 3 gives a detailed case report for this female patient. This summary figure shows only the 3rd, 50th and 97th percentile lines for Tanner II-IV girls as an example but due to the limitation of space, the full spectrum of race/ethnicity–nonspecific curves for girls and boys are found in Appendix Figures 3 through 12 [26]. Using these TSA_Height_ charts (Appendix Figs 3-42) [26], a clinician can plot a child’s height according to their chronological age and Tanner stage to guide clinical management and parent/guardian discussion. Percentile ranges for race/ethnicity–nonspecific and race/ethnicity–specific curves are provided in Appendix Tables 1 to 8 [26]. We developed a clinical online calculator (https://tsaheight2020.shinyapps.io/tsa_height_clinical_calc_plotter_2020/) for determining TSA_Height_ Z scores (TSA_HAZ_). The calculator and TSA_Height_ growth charts including race/ethnicity–specific curves are available at: https://tsaheight2020.shinyapps.io/tsa_height_clinical_calc_plotter_2020; http://doi.org/10.17605/osf.io/myur7; https://www.pediatrics.umn.edu/divisions/endocrinology/patients-families/center-cah-and-dsd/growth.

## Discussion

We have developed specialized TSA_Height_ charts, reference ranges, and an online Z-score calculator (R-shiny APP) using expansive US collated Tanner stages and anthropometry data of US youths from multiple race/ethnicity groups spanning several decades. The application of this method of adjustment of pubertal status on attained height in a large national sample is important because it has become difficult to collect pubertal status in large populations studies in healthy children due to religious, cultural, and child safety concerns related to the performance of physical assessments of pubertal status. Although differences in pubertal timing have been noted among US youth for decades, growth charts accounting for pubertal timing have yet to be established [22]. Furthermore, as puberty progresses, changes in HPG hormones, such as testosterone, luteinizing hormone, estrogen, and inhibin B, track with age and Tanner staging [33]. Increases in these HPG hormones, coupled with growth-promoting hormones (eg, growth hormone, insulin like growth factor-I), influence the changes in linear growth and body composition that occur during puberty. Therefore, pubertal maturation–adjusted growth charts may better represent these hormonally dependent somatic changes compared with chronological age–conditioned reference data [13, 22], which do not capture these changes [16]. Moreover, despite the recommendation for the use of multiethnic sampling in the development of such reference charts [18], more robust data from a large ethnic group from the HHANES were not included in the US CDC 2000 CA_Height_ growth chart development [22]. Similarly, from a global perspective, the current World Health Organization (WHO) 2007 charts for school age youth 5 to 19 years of age exclude weight-for-age data after 10 years due to puberty [13, 18, 34]. Combined, these findings suggest that both the 5- to 19-year WHO and the 2- to 20-years CDC 2000 (CA_Height_) growth charts may not be adequate for clinical practice in adolescents actively progressing through puberty. Analysis of longitudinal data with multiple anthropometric measures within a pubertal maturation stage and incorporating age, would allow better modeling than cross-sectional studies like the NHANES could further extend the usage of this contribution. Nonetheless, as per American Academy of Pediatrics recommendations, clinicians in the United States use the CDC 2000 height charts to longitudinally assess linear growth even though they were generated from cross-sectional data. Hence, our TSA_Height_ charts offers direct clinical utility and applicability as it aligns with the main tool that is currently used for clinical care and management.

In clinical practice, plotting a child’s height on the TSA_Height_ chart (Appendix Figs 3-42) [26] provides a direct visual assessment of height ranking relative to a national sample. This could be useful to patients and their families, and for the clinicians to determine whether a child’s growth is appropriate for pubertal status. The TSA_Height_ charts represent children at early, average, and late puberty and the curves reflect this height distribution within each pubertal maturation stage. These specialized charts are therefore particularly well suited for critically assessing linear growth of children with early puberty that are tall for their age and those with late puberty that are short for their age. Providing race/ethnicity–nonspecific and race/ethnicity–specific charts and data tables similar to those for CDC 2000 CA_Height_ curves available would allow the clinician to plot the height of children irrespective of race/ethnicity group and use the chart that makes the most sense for the child. In children with normal variants of puberty, including “early” and “late” puberty, the use of TSA_Height_ charts may provide reassurance to the treating clinician by demonstrating that the growth response is appropriate for their pubertal status. As we have demonstrated in Fig. 3, it is possible to follow a child longitudinally on the relevant chart while they remain in a single Tanner stage. Once puberty advances, the child would need to be evaluated on the relevant chart for the new Tanner stage. Our expectation is not that the TSA_Height_ curves replace the use of CA_Height_ curves but be supportive tools for assessing children who have early or late puberty that would impact the interpretation of their linear growth patterns. In children with chronic disease, such as inflammatory bowel disease, systemic arthritis, chronic renal insufficiency, and severe asthma, the use of the TSA_Height_ charts may be helpful in monitoring whether children undergoing treatment for these conditions have normal growth or catch-up growth during each pubertal maturation stage.

These growth charts may also be useful in pediatric endocrine practice, for example when monitoring children requiring GnRH analogues to suppress precocious puberty or children with primary or secondary gonadal failure who require hormonal therapy for puberty induction. In such situations, the growth pattern is often different from chronological age–related (CA_Height_) growth charts. An example of the clinical use of TSA_Height_ adjustment to monitor growth during puberty suppression in a child with congenital adrenal hyperplasia and precocious puberty is illustrated in Fig. 3. Our TSA_Height_ growth charts, reference percentiles, and Z-score calculator can also be used in the diagnostic evaluation for growth hormone deficiency in children with a history of cancer therapy, precocious puberty, or constitutional delay of growth and puberty. Since adjustment of insulin-like growth factor 1 levels for pubertal status has been shown to have a better positive predictive power for diagnosing growth hormone deficiency [35], TSA_Height_ adjustment may improve diagnostic accuracy in children with these conditions.

A major strength of our study is that the large amount of data we used to develop the TSA_Height_ reference range came from a 30-year period and incorporated data from many of the same children used to develop the CDC 2000 and WHO 2007 height-for-chronologic age reference charts used globally. Our inclusion of children of diverse race/ethnic backgrounds, including the predominant Hispanic ethnicities in the US—Mexican, Cuban, and Puerto Rican Americans—strengthens the race/ethnicity–specific and race/ethnicity–nonspecific TSA_Height_ data, allowing more general applicability of this adjustment to other population groups.

Using longitudinal growth data including assessment of pubertal maturation would provide the most accurate description of linear growth as children progress through puberty. However, the majority of the available longitudinal linear growth models are based on mathematical expressions of height (y-axis) against time (age) and do not incorporate pubertal status. As such, models are unable to incorporate functional/biological milestones like Tanner stages and model predictions and parameters (eg, velocity at peak height velocity), are only proxies of actual biologic progress. Although the cross-sectional nature of the study does not allow us to assess the duration that a child is in a pubertal stage, tempo of puberty, or height velocity, in our analysis, we sought to characterize the height-for-age ranking of children in a given Tanner stage (“in stage”) which is possible to achieve using cross-sectional data. Thus, the cross-sectional nature of the data is not a limitation for the purposes of this study.

Another strength of our study is that the adjustment for pubertal status may be applicable to datasets that don’t include pubertal assessment. Since we are able to demonstrate how pubertal maturation adjustment impacts the current CDC height-for-age curve, mathematical prediction equations could be used to estimate pubertal maturation related coefficients which could then be applied to a new set of data that does not include pubertal maturation assessment. These prediction equations can be used to calculate TSA height–SDS so as to estimate the impact of pubertal maturation status in a cohort without those data available, similar to what’s been done in prior research concerning insulin-like growth factor 1 and bone mineral density SDS in youths in 3 different countries [35-38]. This is relevant, since it has become difficult to collect pubertal status in large population-based studies in healthy children, including in NHANES, for a variety. The curves generated by our modeling coupled with the sensitivity analysis shows that the group with pubertal assessment appears to be on average older (~1 year), taller, and higher BMI than the CDC 2000 population at younger ages. These differences could stem from the sampled population as we utilized data of children in the HHANES who were predominantly Mexican American, generally older (Tanner staging conducted ages 10 years+) and have previously been shown to be heavier and shorter [39, 40]. There may also be a bias towards taller children in families willing to participate in a study including pubertal assessment. A potential weakness of our study is that our data predates the existing more contemporary clinic-based pubertal datasets [41, 42]. Recent studies in European and Nordic countries have demonstrated marked secular trends in height in addition to earlier onset of puberty over recent decades [43, 44]. However, we believe similar secular trends in height may not have occurred among US children. If such trends occurred in our study population, it would be expected to affect both the pubertal timing and attained stature as previously shown [45]. In fact, we did not observe birth cohort secular trends in height in our study (data not shown).

## Conclusion

Our new race/ethnicity–nonspecific and race/ethnicity–specific TSA_Height_ charts, reference tables, and Z-score calculator accounting for race/ethnicity and pubertal status provide much-needed tools for clinicians to assess and manage linear growth potential for US children over the course of pubertal progression. For clinical researchers, the TSA_Height_ reference charts, tables, and programming codes may be used to apply this adjustment approach to study populations with available pubertal status.

## Abbreviations

CA: chronological age
CA_Height_: height-for-chronological age
CDC: Centers for Disease Control and Prevention
HPG: hypothalamic-pituitary-gonadal
HIS: Hispanic American
NHB: Non-Hispanic Black
NHW: Non-Hispanic White
BMI: body mass index
HHANES: Hispanic Health and Nutrition Examination Surveys
NHANES: National Health and Nutrition Examination Surveys
NHES: National Health Examination Surveys
SDS: standard deviation score
TSA_Height_: Tanner stage–adjusted height-for-age

## Additional Information

***Disclosure Summary:*** Authors have no conflicts of interest to disclose. Dr Addo has no financial relationships to disclose. Dr Miller is a consultant for Abbvie, Ascendis, BioMarin, Bluebird Bio, Novo Nordisk, Pfizer, Sandoz, Sanofi Genzyme, and Tolmar and has received research support from Alexion, Abbvie, Amgen, Ascendis, BioMarin, Novo Nordisk, Opko, Protalix, Sandoz, Sangamo, Sanofi Genzyme, and Takeda. Dr Sarafoglou receives research support from the DHHS Federal Food and Drug Administration, NIH National Cancer Institute, National Science Foundation, Spruce Biosciences, Alexion and Neurocrine.

***Author Contributions:*** Dr Miller: Developed research concept and design, contributed to manuscript write-up, interpretation and made critical revisions for intellectual content.

Dr Sarafoglou: Contributed to research concept and design, manuscript write-up, interpretation, made critical revisions for intellectual content. Dr Addo: Developed research concept and design, performed statistical analyses, manuscript write-up, interpretation made critical revisions for intellectual content. All authors approved the final manuscript as submitted and agree to be accountable for all aspects of the work.

***Data Availability:*** All data generated or analyzed during this study are included in this published article or in the data repositories listed in References.
